# A Retrospective Study of the Relationship Between the Triglyceride Glucose Index and Myocardial Revascularization for New-Onset Acute Coronary Syndromes

**DOI:** 10.3389/fcvm.2022.862252

**Published:** 2022-03-24

**Authors:** Jiatian Li, Yajuan Lin, Han Li, Rui Fan, Li Lin, Xinying Wang, Yinong Jiang, Yun-Long Xia, Bo Zhang, Xiaolei Yang

**Affiliations:** ^1^Institute of Cardiovascular Diseases, The First Affiliated Hospital of Dalian Medical University, Dalian, China; ^2^Department of Cardiology, The First Affiliated Hospital of Dalian Medical University, Dalian, China

**Keywords:** TyG index, serum uric acid, myocardial revascularization, acute coronary syndromes, coronary artery disease

## Abstract

**Background:**

This study explored the relationship between the TyG index/serum uric acid (SUA) panel and myocardial revascularization (MRT) for new-onset acute coronary syndromes (ACS).

**Methods:**

Between January 2011 and July 2020, 13,271 new-onset ACS patients were enrolled. The logistic regression models and the odds ratios (ORs) were used to quantify the risk of TyG index/SUA and MRT. Then, interaction analyses of TyG index and SUA on MRT were applied.

**Results:**

Elevated TyG index was positively associated higher risks of MRT. However, SUA levels were negatively associated with MRT. Compared with those in the lowest quartile, the risk of MRT increased gradually among patients in Q1 of the SUA category (OR = 1.03, 1.11, and 1.28 for Q2, Q3, and Q4 of TyG index, respectively), Q2 of the SUA category (OR = 1.41, 1.68, and 2.18 for Q2, Q3, and Q4 of TyG index, respectively), Q3 of the SUA category (OR = 1.05, 1.45, and 1.45 for Q2, Q3, and Q4 of TyG index, respectively), and Q4 of the SUA category (OR = 1.20, 1.29, and 1.46 for Q2, Q3, and Q4 of TyG index, respectively). This pattern was observed in both male and female, as well as patients without type 2 diabetes mellitus.

**Conclusion:**

Patients with a higher TyG index have a higher proportion of MRT in new-onset ACS. This result also applies to patients with different levels of SUA during new-onset ACS.

## Introduction

Coronary artery disease (CAD), a multifactorial fatal disease, has a high global prevalence. According to the latest heart disease statistics published in 2021 (1), an estimated 20.1 million Americans ≥ 20 years old had CAD. Although superior evidence-based strategies such as optimizing drug treatment and revascularization have been widely developed and applied in recent years, CAD patients, especially those who have suffered from acute coronary syndromes (ACS), continue to be at high risk for adverse cardiovascular deaths. The gold standard for ACS diagnosis is invasive coronary angiography. When coronary angiography shows that the stenosis has reached 75% or more of the diameter of the coronary lumen, patients will be advised to undergo myocardial revascularization (MRT) (2). A risk-factor assessment is critical for ACS patients, and insulin resistance (IR) has been considered to be a prognostic predictor of CAD.

Insulin resistance occurs when tissues are not sufficiently sensitive to the effects of insulin despite normal or, more often, elevated levels. Hyperinsulinaemic-euglycaemic clamp is the gold standard technique for IR. However, it has limitations in clinical application due to the complexity of detecting technology. The requirement to measure fasting insulin level also limits the homeostasis model assessment of IR (HOMA-IR) as a common clinical testing method. The triglyceride-glucose index (TyG index) is a new IR indicator, and it is calculated from fasting triglyceride (TG) and fasting plasma glucose (FPG). The TyG index is significantly associated with IR measured by the hyperinsulinaemic-euglycaemic clamp, and it outperforms HOMA-IR in non-diabetic and diabetic patients (3, 4). Previous studies have linked the TyG index to atherosclerotic cardiovascular diseases such as carotid atherosclerosis (5), arterial stiffness (6), adverse cardiovascular events in diabetes (7), and poor prognosis in patients undergoing percutaneous coronary intervention (PCI) (8).

Serum uric acid (SUA), an ultimate product of purine metabolism, has been associated with the pathogenesis of many cardiovascular diseases. For example, Niu et al. (9) discovered a relationship between SUA and hypertension in Chinese population, while Chen et al. (10) found that high levels of SUA are significantly related to the prevalence of atrial fibrillation. Furthermore, SUA has been associated with chronic kidney diseases, heart failure, and cardiovascular death (11). However, to our knowledge, there is no study on the interaction between TyG index with various levels of SUA in MRT for new-onset ACS. Therefore, this study aimed to explore the relationship between the TyG index/SUA panel and MRT in new-onset ACS, as well as whether TyG index predicts the risk of MRT in new-onset ACS patients.

## Materials and Methods

### Patients and Data Source

**FIGURE 1:**
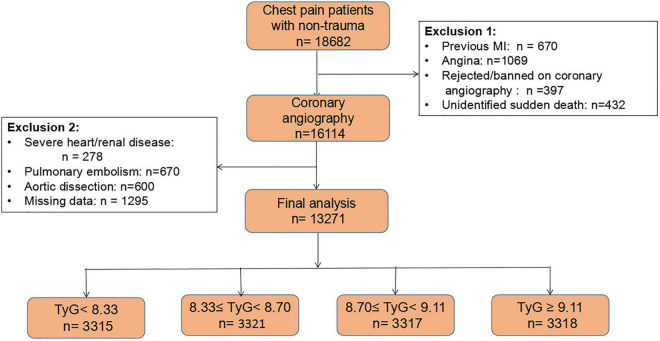
The flow chart of this cohort study.

This was a cross-sectional study. New-onset ACS patients were enrolled for this study from Chest Pain Center and Heart Center of the First Affiliated Hospital of Dalian Medical University in northern China between January 2011 and July 2020. The inclusion criteria were as follows: (1) all patients had to be over 18 years old; and (2) patients had chest pain and with no previous history of PCI or coronary artery bypass graft (CABG). At the beginning of data analysis, we included 18,682 adults who had chest pain without traumatic factors. Patients were excluded if they: (1) had myocardial infarction (MI) (*n* = 670); (2) had angina (*n* = 1,069); (3) refused or were not suitable for coronary angiography (*n* = 397); or died unexpectedly (*n* = 432). At this stage, 16,114 patients who underwent coronary angiography were retained. Furthermore, patients with severe diseases such as structural heart/renal disease (*n* = 278), pulmonary embolism and aortic dissection (*n* = 1,270) were excluded. Lastly, those with incomplete data (*n* = 1,295) were excluded. Accordingly, the final dataset contained 13,271 patients. The flow chart of the study is shown in Figure 1. The study protocol was approved by the First Affiliated Hospital of Dalian Medical University. All procedures were performed in line with the declaration of Helsinki and its amendments, and all participants provided written informed consent to participate in this study.

### Coronary Angiography and Myocardial Revascularization

Coronary angiography was performed in all 13,271 patients between January 1st 2011 and July 31st 2020. All coronary angiographies were conducted by qualified physicians and nurses, and measurements were recorded using digital subtraction angiography. We followed the ESC/EACTS guidelines while deciding whether to perform MRT. Stenosis was considered severe when its diameter in at least one of the major coronary artery branches [Left coronary artery (LCA), left anterior descending (LAD), left circumflex (LCX), and right coronary artery (RCA)] was ≥75%. In according with the hospital protocol, two qualified cardiologists reviewed the coronary angiography results. If the two cardiologists disagreed each other, a senior interventional cardiologist was consulted, to ensure correct assessment prior to final analysis.

### Data Collection and Definitions

Age, gender, height, weight, history of previous PCI, and previous CABG data were obtained from medical records by trained cardiologists. For acute ST-segment elevation myocardial infarction (STEMI) requiring expeditious MRT, some indicators were obtained after admission and before revascularization, such as SUA, creatinine, red blood cell count (RBC), platelet (PLT), and white blood cell count (WBC). Some of the indicators were obtained after fasting overnight, such as FPG, TG, total cholesterol (TC), low-density lipoprotein cholesterol (LDL-C), high-density lipoprotein cholesterol (HDL-C) and lipoprotein(a) [Lp(a)]. For acute non-ST-segment elevation myocardial infarction (NSTEMI), all indicators were collected after an overnight of fasting after hospitalization. Body mass index (BMI) was calculated by dividing weight (kg) by height (m^2^). The TyG index was calculated as the ln (fasting TG level [mg/dL] × FPG level [mg/dL]/^2^). Participants were prohibited from smoking and consuming caffeinated beverages or alcohol prior to taking their blood pressure records. They were then asked to remain seated for at least 30 mins. Hypertension was characterized as systolic blood pressure (SBP) at ≥140 mmHg and/or diastolic blood pressure (DBP) at ≥90 mmHg, or a self-reported history of hypertension and the patient was using anti-hypertensive drugs. Type 2 diabetes mellitus (T2DM) was defined as FPG ≥ 7.0 mmol/L or a self-reported history of T2DM. Current smokers were defined those who had a recent history of smoking, or reported cigarettes consumption >100 cigarettes in total. Drinking status were self-reported and classified as never drinkers, past drinkers, or current drinker.

### Statistical Analyses

For statistical description, when continuous variables proved to be normally distributed, they were expressed as mean ± standard deviation, and as median and interquartile range (IQR) when they are not normally distributed. Categorical variables are reported as frequencies with percentages. The participants were stratified into four quartiles based on their TyG index. The distribution of baseline demographic characteristics, laboratory data, MRT and branches of coronary atherosclerosis was compared between groups, and for comparing methods we used one-way ANOVA for continuous variables between groups, and a Chi square test or Fisher’s exact test for categorical variables between groups. The logistic regression models were used to analyze the relationship between TyG index and MRT. The risk associated with TyG index and MRT was assessed using the odds ratio (OR) with 95% confidence interval (CI), with the lowest quartile serving as the reference. We performed two models: (a) adjusted for age and gender; and (b) adjusted for age, gender, BMI, SBP, SUA, creatinine, HDL-C, TC, RBC, WBC, PLT, statin use, current smoker, current drinker, diabetes, and hypertension. In the sensitivity analysis, we re-completed the above analysis in non-diabetes patients. A two-sided analysis with a *P* value < 0.05 was considered significant. All of the analyses were performed using SPSS version 23.0 (IBM Corp, Armonk, NY, United States).

## Results

### Baseline Characteristics of the Patients

The baseline characteristics of the patients are shown in Table 1. This study comprised 13,271 patients who were divided into four groups based on the quartile of the TyG index as follows: first quartile (Q1): *n* = 3,315, with TyG index < 8.41; second quartile (Q2): *n* = 3,321, 8.41 ≤ TyG index < 8.80; third quartile (Q3): *n* = 3,317, 8.80 ≤ TyG index < 9.25; and fourth quartile (Q4): *n* = 3,318, TyG index ≥ 9.25. The patients in this study had an average age of 63.02 ± 9.95 years, with 9,443 (71.2%) being male. The mean age in each of the four groups was 61.04 ± 9.18 (Q1), 62.07 ± 8.61 (Q2), 62.51 ± 9.02 (Q3), and 63.21 ± 10.74 (Q4). There were significant differences among the four groups in terms of SBP, BMI, TC, HDL-C, TG, FPG, SUA, creatinine, RBC, WBC, PLT, current drinker, current smoker, T2DM, hypertension and MRT, but no significant differences were detected in the other indicators. Patients in the fourth quartile had the highest distribution of coronary atherosclerosis in LCA, LAD, LCX, and RCA branches (*P* < 0.05), which showed in Table 1.

**TABLE 1:** Baseline clinical characteristics of patients in different triglyceride-glucose (TyG) index groups.

Variables	TyG < 8.41 mg/dl *n* = 3,315	8.41 ≤ TyG < 8.80 mg/dl *n* = 3,321	8.80 ≤ TyG < 9.25 mg/dl *n* = 3,317	TyG ≥ 9.25 mg/dl *n* = 3,318	*P* Valve
Age (year)	61.04 ± 9.18	62.07 ± 8.61	62.51 ± 9.02	63.21 ± 10.74	<0.001
Male, *n* (%)	2,322 (70.0%)	2,375 (71.5%)	2,324 (73.0%)	2,422 (73.0%)	0.035
SBP (mmHg)	128.50 (118.0, 145.0)	131.8 (118.0, 150.0)	136.9 (119.0, 155.0)	139.0 (120.0, 156.0)	0.038
BMI (kg/m^2^)	26.16 ± 3.68	26.17 ± 3.69	26.24 ± 3.70	26.95 ± 3.73	0.048
LDL-C (mmol/L)	2.44 (1.86, 2.96)	2.45 (1.87, 2.95)	2.46 (1.88, 2.96)	2.50 (1.87, 2.98)	0.191
TC (mmol/L)	4.02 (3.37, 4.87)	4.27 (3.58, 5.08)	4.47 (3.73, 5.32)	5.33 (4.09, 6.08)	<0.001
HDL-C (mmol/L)	1.00 (0.91,1.28)	1.05 (0.90, 1.66)	1.15 (0.90, 1.86)	1.19 (1.10, 2.24)	<0.001
LP(a) (mmol/L)	161.0 (85.0, 309.0)	161 (86.0, 312.7)	156.8 (86.5, 299.0)	160.60 (87.0, 305.9)	0.96
TG (mmol/L)	0.84 (0.70, 0.99)	1.28 (1.11,1.46)	1.79 (1.46, 2.09)	2.81 (2.04, 4.09)	<0.001
FPG (mmol/L)	4.94 (4.57, 5.40)	5.25 (4.79, 5.96)	5.61 (4.98, 6.91)	6.68 (5.23, 9.72)	<0.001
SUA (mmol/L)	305.3 (269.0, 398.0)	326.5 (271.0, 406.0)	348.1 (285.0, 423.0)	355.8 (289.0, 440.0)	<0.001
Creatinine (mmol/L)	69.00 (59.86, 79.00)	71.24 (60.00, 79.00)	72.60 (60.01, 80.00)	72.88 (61.0, 81.0)	0.046
WBC (10^∧^9/L)	6.49 (5.26, 8.06)	6.81 (5.60, 8.41)	6.86 (5.56, 8.57)	7.56 (5.68, 8.86)	<0.001
Platelet (10^∧^9/L)	198.0 (164.0, 242.7)	203.0 (169.0, 243.0)	214.0 (173.0, 246.0)	218.6 (178.0, 251.0)	0.005
RBC (10^∧^9/L)	4.00 (4.14, 4.79)	4.05 (4.15, 4.90)	4.35 (4.15, 4.99)	4.59 (4.17, 5.10)	<0.001
Current smoker, *n* (%)	1,410 (42.5%)	1,378 (41.5%)	1,378 (41.5%)	1,337 (40.3%)	0.042
Current drinker, *n* (%)	760 (22.9%)	731 (22.0%)	737 (22.2%)	666 (20.1%)	0.009
Statin use, *n* (%)	2,801 (84.6%)	2,766 (83.3%)	2,752 (83.0%)	2,770 (83.5%)	0.197
HTN, *n* (%)	1,236 (37.3%)	1,529 (46.0%)	1,628 (49.1%)	1,665 (50.2%)	<0.001
Diabetes, *n* (%)	309 (9.3%)	560 (16.9%)	888 (26.8%)	1,456 (43.9%)	<0.001
MRT, *n* (%)	1,772 (53.5%)	2,044 (61.5%)	2,155 (65.0%)	2,230 (67.2%)	<0.001
**Distribution of coronary atherosclerosis**					
LCA, *n* (%)	295 (8.9%)	343 (10.3%)	368 (11.1%)	370 (11.2%)	0.001
LAD, *n* (%)	2,124 (64.1%)	2,336 (70.3%)	2,403 (72.4%)	2,502 (75.4%)	<0.001
LCX, *n* (%)	1,555 (46.9%)	1,626 (49.0%)	1,822 (54.9%)	1,928 (58.1%)	<0.001
RCA, *n* (%)	1,791 (54.0%)	1,979 (59.6%)	2,073 (62.5%)	2,129 (64.2%)	<0.001

*BMI, body mass index; FPG, fasting plasma glucose; HDL-C, high density lipoprotein cholesterol; HTN, hypertension; LDL-C, low-density lipoprotein cholesterol; MRT, myocardial revascularization; RBC, red blood cell count; SBP, systolic blood pressure; SUA, serum uric acid; WBC, white blood cell count; TC, total cholesterol; TG, triglyceride; LCA, left coronary artery; LAD, left anterior descending; LCX, left circumflex artery; RCA, right coronary artery.*

### The Prevalence of Myocardial Revascularization Increases With Triglyceride-Glucose Index

The prevalence of MRT in the entire population, corresponding to Q1, Q2, Q3, and Q4, was 1,772 (53.5%), 2,044 (61.5%), 2,155 (65.0%), and 2,230 (67.2%). The prevalence of MRT gradually increased as TyG index increased. This phenomenon was observed in both male and female of the total population. The results of univariate and multivariate analysis logistic regression are presented in Table 2. Model 2 indicated that this relationship remained significant, even after adjusted for age, gender, BMI, SBP, SUA, creatinine, HDL-C, TC, RBC, WBC, PLT, statin use, current smoker, current drinker, diabetes, and hypertension. Even after adjusting for multivariate factors, patients with a higher TyG index had a greater risk of MRT. In the whole population, the adjusted OR (95% CI) for patients in Q2, Q3, and Q4 of the TyG index was 1.18 (1.05–1.33), 1.30 (1.16–1.47), and 1.55 (1.37–1.75), respectively, when compared to Q1 (*P* < 0.001), and this trend is meaningful between groups (*P* for trend: *P* < 0.001). In the male, the adjusted OR (95% CI) for patients in Q2, Q3, and Q4 were 1.12 (1.01–1.28), 1.26 (1.10–1.46), and 1.45 (1.25–1.68), respectively, when compared to first quartile (*P* < 0.001). In females, the adjusted OR (95% CI) for patients in Q2, Q3, and Q4 was 1.31 (1.05–1.64), 1.37 (1.06–1.68), and 1.59 (1.23–2.06), respectively.

**TABLE 2:** The risk estimate for the myocardial revascularization based on the TyG quartiles.

	Q1	Q2	Q3	Q4	*P* for trend
**Total**					
Myocardial revascularization, *n* (%)	1,772 (53.5%)	2,044 (61.5%)	2,155 (65.0%)	2,230 (67.2%)	
Model 1	Ref.	1.31 (1.18, 1.45)	1.57 (1.42, 1.74)	2.08 (1.87, 2.31)	<0.001
Model 2	Ref.	1.18 (1.05, 1.33)	1.30 (1.16, 1.47)	1.55 (1.37, 1.75)	
**Male**					
Myocardial revascularization, *n* (%)	1,281 (55.2%)	1,476 (62.1%)	1,513 (65.1%)	1,617 (66.8%)	
Model 1	Ref.	1.25 (1.11, 1.41)	1.48 (1.31, 1.68)	1.90 (1.68, 2.15)	<0.001
Model 2	Ref.	1.12 (1.01, 1.28)	1.26 (1.10, 1.46)	1.45 (1.25, 1.68)	
**Female**					
Myocardial Revascularization, *n* (%)	491 (49.4%)	568 (60.0%)	642 (64.7%)	613 (68.4%)	
Model 1	Ref.	1.48 (1.12,1.80)	1.81 (1.49, 2.20)	2.57 (2.09, 3.17)	<0.001
Model 2	Ref.	1.31 (1.05, 1.64)	1.37 (1.06, 1.68)	1.59 (1.23, 2.06)	

***Models 1:** adjusted for age, gender.*

***Models 2:** adjusted for age, gender, BMI, SBP, SUA, creatinine, HDL-C, TC, RBC, WBC, PLT, statin use, current smoker, current drinker, diabetes, and hypertension.*

*BMI, body mass index; HDL-C, high density lipoprotein cholesterol; PLT, platelets; RBC, red blood cell count; SBP, systolic blood pressure; SUA, serum uric acid; WBC, white blood cell count; TC, total cholesterol.*

### The Prevalence of Myocardial Revascularization Decreases as Serum Uric Acid Increases

Table 3 shows the prevalence and ORs of MRT in onset-ACS patients grouped based on the quartiles of SUA values. The prevalence of MRT in the entire population was 2,487 (70.9%), 2,223 (61.8%), 2,061 (58.2%), and 2,047 (57.2%) in Q1, Q2, Q3, and Q4 of SUA, respectively. The prevalence of MRT gradually decreased as SUA increased. When compared to Q1, the adjusted OR (95% CI) for patients in Q2, Q3, and Q4 compared to Q1 was 0.76 (0.68–0.86), 0.63 (0.51–0.70), and 0.57 (0.51–0.64), respectively (*P* < 0.001) in the total population, and 0.61 (0.53–0.69), 0.60 (0.47–0.68), and 0.58 (0.51–0.70), respectively (*P* < 0.001) in male. A similar trend was observed in female, the adjusted OR (95% CI) for patients in Q2, Q3, and Q4 compared to Q1 was 0.68 (0.59–0.88), 0.50 (0.44–0.67), and 0.47 (0.32–0.65), respectively (*P* < 0.001) in female.

**TABLE 3:** The risk estimate for the myocardial revascularization based on the UA quartiles.

	Q1	Q2	Q3	Q4	*P* for trend
**Total**					
Myocardial revascularization, *n* (%)	2,487 (70.9%)	2,223 (61.8%)	2,061 (58.2%)	2,047 (57.2%)	
Model 1	Ref.	0.68 (0.61,0.76)	0.61 (0.55,0.68)	0.58 (0.52,0.64)	<0.001
Model 2	Ref.	0.76 (0.68,0.86)	0.63 (0.51,0.70)	0.57 (0.51,0.64)	
**Male**					
Myocardial revascularization, *n* (%)	1,691 (72.0%)	1,556 (61.2%)	1,531 (59.3%)	1,557 (58.2%)	
Model 1	Ref.	0.64 (0.57,0.74)	0.62 (0.55,0.70)	0.59 (0.52,0.69)	<0.001
Model 2	Ref.	0.61 (0.53,0.69)	0.60 (0.47,0.68)	0.58 (0.51,0.70)	
**Female**					
Myocardial revascularization, *n* (%)	796 (68.6%)	667 (63.0%)	530 (55.2%)	490 (54.0%)	
Model 1	Ref.	0.77 (0.64,0.93)	0.56 (0.46,0.68)	0.52 (0.43,0.64)	<0.001
Model 2	Ref.	0.68 (0.59,0.88)	0.50 (0.44,0.67)	0.47 (0.32,0.65)	

***Models 1:** adjusted for age, gender.*

***Models 2:** adjusted for age, gender, BMI, SBP, SUA, creatinine, HDL-C, TC, RBC, WBC, PLT, statin use, current smoker, current drinker, diabetes, and hypertension.*

*BMI, body mass index; HDL-C, high density lipoprotein cholesterol; PLT, platelets; RBC, red blood cell count; SBP, systolic blood pressure; SUA, serum uric acid; WBC, white blood cell count; TC, total cholesterol.*

### The Effect of Triglyceride-Glucose Index and Serum Uric Acid Interaction on Myocardial Revascularization

**FIGURE 2:**
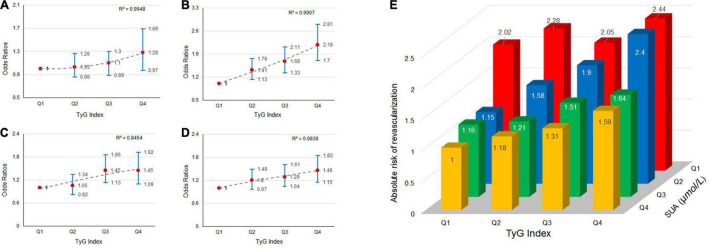
The risk of myocardial revascularization (MRT) in new-onset acute coronary syndromes (ACS) patients based on baseline triglyceride-glucose (TyG) index quartiles. Adjusted odds ratios (ORs) for MRT in: **(A)** SUA-quartiles 1; **(B)** SUA-quartiles 2; **(C)** SUA-quartiles 3; and **(D)** SUA-quartiles 4. **(E)** Absolute risk for MRT in each group based on the TyG index quartiles and SUA quartiles.

**FIGURE 3:**
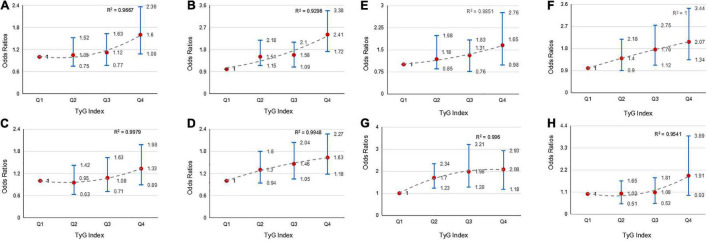
The risk of MRT in males and females based on baseline TyG index quartiles. Adjusted ORs for MRT in males in: **(A)** SUA-quartiles 1; **(B)** SUA-quartiles 2; **(C)** SUA-quartiles 3; and **(D)** SUA-quartiles 4. Adjusted ORs for MRT in females in: **(E)** SUA-quartiles 1; **(F)** SUA-quartiles 2; **(G)** SUA-quartiles 3; and **(H)** SUA-quartiles 4.

**FIGURE 4:**
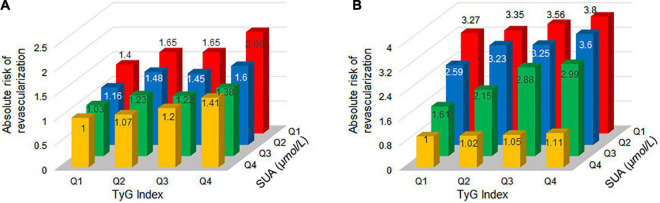
Absolute risk of MRT by TyG index and SUA quartiles for new-onset ACS patients in males and females. Absolute risk of MRT in: **(A)** males and **(B)** females.

We explored the effect of TyG index and SUA interaction in MRT. The risk of MRT increased gradually as TyG index levels increased among patients in first quartile of the SUA category (OR = 1.03, 1.11, and 1.28 for Q2, Q3, and Q4 of TyG index, respectively), second quartile of the SUA category (OR = 1.41, 1.68, and 2.18 for Q2, Q3, and Q4 of TyG index, respectively), third quartile of the SUA category (OR = 1.05, 1.45, and 1.45 for Q2, Q3, and Q4 of TyG index, respectively), and fourth quartile of SUA category (OR = 1.20, 1.29, and 1.46 for Q2, Q3, and Q4 of TyG index, respectively). The ORs associated TyG index in participants grouped by SUA quartiles are showed in Figures 2A–D. Similarly, the patients in the higher TyG index quartiles, had a higher probability of being treated with MRT when grouped based on different SUA quartiles (Figure 2E). Figure 3 shows the results of further subgroup analysis in male and female patients. After adjusting for the different confounding factors, positive associations between TyG index and MRT were found in both male (Figures 3A–D) and female (Figures 3E–H) patients. Figure 4 shows that patients in the highest quartile of TyG index values were at the highest risk of undergoing MRT.

### Sensitivity Analyses

**FIGURE 5:**
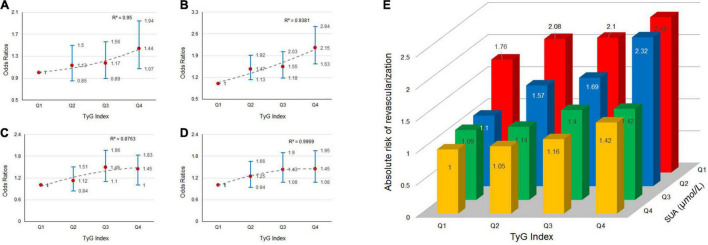
The risk of MRT based on TyG index quartiles grouped by SUA quartiles in new-onset ACS patients without diabetes. Adjusted ORs for MRT in: **(A)** SUA-quartiles 1; **(B)** SUA-quartiles 2; **(C)** SUA-quartiles 3; and **(D)** SUA-quartiles 4. **(E)** Absolute risk for MRT in each group based on the TyG index quartiles and SUA quartiles.

To validate the effect of TyG index level on MRT, we further analyzed the aforementioned method in non-diabetic new-onset ACS patients. Results of sensitivity analyses are shown in Table 4 and Figure 5. It can be found from Table 4 that after excluding diabetic patients, the ORs changed a little, and the relationship between TyG and MRT still established. Figures 5A–D shows the highest ORs (95% CI) for MRT categorized by SUA quartiles were observed in TyG index fourth quartile quartiles [1.44 (1.07, 1.94) for first quartile of SUA, 2.15 (1.63, 2.84) for second quartile of SUA, 1.45 (1.00, 1.83) for third quartile of SUA, and 1.45 (1.08, 1.95) for fourth quartile of SUA]. Figure 5E shows that patients in the highest quartile of TyG index values were at the highest risk of undergoing MRT. Generally, a higher TyG index was significantly associated with a higher incidence of MRT across non-diabetic patients.

**TABLE 4:** The risk of the myocardial revascularization based on the TyG quartiles in non-diabetic patients.

	Q1	Q2	Q3	Q4	*P* for trend
**Total**					
Model 1	Ref.	1.25 (1.11, 1.40)	1.39 (1.23, 1.56)	1.69 (1.50, 1.90)	<0.001
Model 2	Ref.	1.17 (1.03, 1.33)	1.30 (1.14, 1.47)	1.55 (1.36, 1.77)	
**Male**					
Model 1	Ref.	1.24 (1.08, 1.42)	1.34 (1.16, 1.54)	1.65 (1.43, 1.90)	<0.001
Model 2	Ref.	1.18 (1.01, 1.37)	1.28 (1.10, 1.49)	1.54 (1.31, 1.80)	
**Female**					
Model 1	Ref.	1.30 (1.04,1.62)	1.51 (1.21, 1.89)	1.71 (1.35, 2.16)	<0.001
Model 2	Ref.	1.05 (0.80, 1.36)	1.28 (0.98, 1.66)	1.31 (0.99, 1.73)	

***Models 1:** adjusted for age, gender.*

***Models 2:** adjusted for age, gender, BMI, SBP, SUA, creatinine, HDL-C, TC, RBC, WBC, PLT, statin use, current smoker, current drinker, diabetes, and hypertension.*

*BMI, body mass index; HDL-C, high density lipoprotein cholesterol; PLT, platelets; RBC, red blood cell count; SBP, systolic blood pressure; SUA, serum uric acid; WBC, white blood cell count; TC, total cholesterol.*

## Discussion

In 13,271 patients with new-onset ACS of this retrospective study, an elevated TyG index was found to be associated with an increased risk of MRT. To our knowledge, this is the first study to investigate the relationship between the TyG index and MRT at different SUA levels in new-onset ACS patients. However, SUA levels were inversely associated with MRT among new-onset ACS patients. Furthermore, the interaction between TyG index and SUA profile showed that patients in higher quartiles of TyG index grouped by SUA component still had a higher chance of receiving MRT. Similar results were obtained after excluding patients with T2DM.

Previous studies have reported correlation between IR and cardiovascular diseases. Silva et al. (12) found that the TyG index was positively correlated with a higher prevalence of symptomatic CAD. After a 3-year follow-up, Wang et al. (7) reveal that the TyG index is an independent predictor of adverse cardiovascular events. According to Zhao et al. (13) an elevated TyG index is significantly associated with a higher risk of arterial stiffness and nephric microvascular damage in elderly patients. The incidence of coronary artery calcification (CAC) progression in 12,326 asymptomatic Korean adults was 30.6% after a mean 3.3 years. The incidence of CAC progression was markedly elevated with higher TyG index, especially in patients without heavy baseline CAC (14). Furthermore, Luo et al. (8) showed that a higher TyG index was correlated with an increased risk of future cardiovascular diseases in STEMI patients undergoing PCI. In a large prospective cohort study from northern China, Tian et al. (15) observed that the TyG index is associated with MI risk, even in people with normal FPG and TG levels. After a median follow-up of 11.03 years, participants in the fourth quantile of the baseline and updated average TyG index, as well as those with threefold higher TyG index, were at a higher risk of MI than other groups. These studies suggest that TyG index is closely related to CAD.

In general, FPG values mainly reflect IR from the liver, whereas fasting TG levels primarily reflect IR from fat cells. Therefore, the TyG index can reflect IR from two aspects. Moreover, the TyG index has been associated with hypertension, dyslipidemia, hyperglycemia, and metabolic syndrome, all of which are risk factors for CAD (16–19). What’s more, it can be explained from the IR represented by the TyG index itself. IR is known to be the major pathophysiologic factors driving T2DM. As a heterogeneous syndrome, T2DM is an independent risk factor for CAD and affects the prognosis. In recent years, prediabetes has been proposed as a separate concept (20), it is defined by FPG is higher than normal level but lower than the thresholds for T2DM. Results indicated that prediabetes was associated with an increased risk of CAD, heart failure and all-cause death in the general population (21, 22). As an early indicator to identify risk factors for CAD, the TyG index can reflect IR in the general population during prediabetes. This is beneficial in providing preventive measures in the event of elevated FPG but below the diagnosis of T2DM.

The mechanism underlying the correlation between TyG index and CAD remains unknown. According to Valeska et al. (23), IR mainly promotes the development of CVD via atherosclerotic plaque formation, ventricular hypertrophy, and diastolic abnormalities. Furthermore, IR is significantly correlated with endothelial dysfunction, oxidative stress, cardiovascular remodeling, coagulation imbalance, and inflammation (23, 24). Glycolysis accounts for less than 10% of total ATP synthesis in a healthy heart for cardiomyocytes. Insulin stimulates glucose uptake by inducing the translocation of glucose transporters type 4 (GLUT4) from intracellular storage sites to the plasma membrane, where the transporter facilitates glucose diffusion into cell (25). When IR occurs, the transformation ability of myocardial cells is impaired, and fatty acids become the only source of energy. Lipotoxicity occurs when fatty acids increase excessively in cardiomyocytes (26). Lipid buildup can potentially cause apoptosis by impairing mitochondrial function, cardiac hypertrophy, and contractile dysfunction (27, 28). These may serve as potential mechanisms to explain the relationship between TyG index and CAD, and undoubtedly this requires more studies.

Serum uric acid, a byproduct of purine metabolism, is a natural antioxidant. The relationship between SUA and CADs has been hotly debated, Zand et al. (29) thought SUA is not an independent risk factor for CAD. However, the study had only 473 patients and was mostly middle-aged (age < 45 years for male and <55 years for female). In large cohort study with decades of follow-up, SUA has been shown to be an independent risk factor for HTN (30), atrial fibrillation (31), and CAD (32, 33). What’s more, Çanga et al. (34) found SUA levels were correlated with long-term major adverse cardiovascular events (MACEs) during long-term follow-up in NSTEMI, which suggested that SUA is a risk factor for CAD. In this study we found that ACS patients with higher SUA had a lower risk of MRT. As a classic antioxidant, this phenomenon may be associated with the status of acute stress. It was reported that SUA has a protective effect on prognosis in acute ischemic events (35, 36). Acute oxidative stress changes when an acute ischemic event occurs. In this case, the subsequent SUA concentration is elevated due to ischemic tissue metabolism of adenosine (37, 38), nitric oxide-induced loss of xanthine oxidase inhibition (39), and impaired oxidative metabolism (40) are similar findings. Therefore, an increase in SUA could be a protective measure against the adverse effects of free radical activity and oxidative stress. Acute increase in SUA may provide short-term protection to ACS patients.

As for the mechanism between SUA and IR, SUA induces IR by inhibiting insulin signaling by reducing the phosphorylated Akt levels in adipose tissue and skeletal muscle, while promoting fat accumulation and glucose production in liver cells (41). Accordingly, SUA enhances lipogenesis by activating ATP-citrate lyase and inhibiting both mitochondrial aconitase activity and AMP-activated kinase (AMPK) phosphorylation, thus predisposing to hepatic steatosis. Furthermore, SUA activates hepatic gluconeogenesis, increases glucose production and aggravates hyperglycemia by reducing AMPK activation (42). SUA is a potent antioxidant that can neutralize superoxide and hydroxyl free radicals in the plasma. However, it can also act as a powerful pro-oxidant by increasing the production of reactive oxygen species (ROS), thereby promoting lipid and glucose metabolism disorders (43). Furthermore, SUA-induced oxidative stress has been reported to induce adipose tissue dysfunction in adipocytes, which subsequently leads to pro-inflammatory endocrine imbalances, resulting in low-grade inflammation and IR (44, 45).

More importantly, more evidence has emerged in recent decades showing that SUA levels have increased during the prediabetes period, indicating that SUA plays a role in the pathogenesis of T2DM (46). Binh et al. (47) discovered significant correlation between hyperuricemia and isolated impaired fasting glucose (IFG), isolated impaired glucose tolerance (IGT), combined IFG-IGT, and T2DM independent of the confounding factors in a Vietnamese population. After a 7.5-year follow-up period, a Rotterdam prospective cohort study (10) found that SUA is a risk factor for prediabetes and T2DM, and that SUA is more closely associated with early-phase mechanisms in the development of T2DM than late-phase mechanisms. Overall, hyperuricemia is associated with prediabetes and contributes to the development of diabetes. One of the characteristics of diabetes and prediabetes is IR. As a new indicator of IR, the TyG index effectively reflects the IR during prediabetes. And the prediabetes is considered a risk factors for CAD (21, 22). In our study, we found the rate of MRT increased with increasing the TyG index. Due to the severe IR in diabetes, we excluded the diabetes in the sensitivity analysis. Similar results were found in the non-diabetic population.

One of the strengths of this study was the large number of new-onset ACS patients who were adjusted to minimize residual confounding factors. The detection of SUA and TyG index would be useful for further risk stratification in prediabetes. The increase of the TyG index is beneficial to provide early preventive measures for prediabetes, before the elevated FPG reaches the threshold of T2DM. However, this research has certain limitations. First, this was a single-center study. Furthermore, the exclusion criteria were narrow, which reduced the generalizability of the study. Thirdly, historical TyG index data of the patients was missing, resulting in an inability to dynamically respond to IR. Finally, our study lacked direct test for IR, due to the high cost and complex detection methods, insulin levels are rarely detected. Therefore, further research is needed to confirm whether the discovered relationship exists among other ethnic groups.

## Conclusion

Patients with a higher TyG index have a higher proportion of myocardial revascularization in new-onset ACS. This result also applies to patients with different levels of SUA during new-onset ACS.

## Data Availability Statement

The raw data supporting the conclusions of this article will be made available by the authors, without undue reservation.

## Ethics Statement

The studies involving human participants were reviewed and approved by The First Hospital of Dalian Medical University Institutional Review Board approved this study. The patients/participants provided their written informed consent to participate in this study.

## Author Contributions

JL and YL contributed to the data collection and analysis and wrote the manuscript. HL, RF, LL, and XW were responsible for the collection of patient information. Y-LX contributed to provide the funding. BZ and XY were responsible for all the manuscript. All authors contributed to the article and approved the submitted version.

## Conflict of Interest

The authors declare that the research was conducted in the absence of any commercial or financial relationships that could be construed as a potential conflict of interest.

## Publisher’s Note

All claims expressed in this article are solely those of the authors and do not necessarily represent those of their affiliated organizations, or those of the publisher, the editors and the reviewers. Any product that may be evaluated in this article, or claim that may be made by its manufacturer, is not guaranteed or endorsed by the publisher.

## Abbreviations

BMI: body mass index
FPG: fasting plasma glucose
HDL-C: high density lipoprotein cholesterol
HTN: hypertension
LDL-C: low-density lipoprotein cholesterol
MRT: myocardial revascularization
RBC: red blood cell count
SBP: systolic blood pressure
SUA: serum uric acid
WBC: white blood cell count
TC: total cholesterol
TG: triglyceride
LCA: left coronary artery
LAD: left anterior descending
LCX: left circumflex artery
RCA: right coronary artery.
